# Primary duodenal tuberculosis complicated with perforation: A review of literature and case report

**DOI:** 10.1016/j.amsu.2021.102392

**Published:** 2021-05-12

**Authors:** Atri Souhaib, Houcine Magherbi, Ouadi Yacine, Anis Hadad, Zehani Alia, Youssef Chaker, Montasser Jamel Kacem

**Affiliations:** a Department of Digestive Surgery ‘A’, La Rabta Hospital University, Faculty of Medicine of Tunis, Tunisia; b Department of Pathological Anatomy, La Rabta Hospital University, Faculty of Medicine of Tunis, Tunisia

**Keywords:** Intestinal tuberculosis, Duodenal tuberculosis, Digestive perforatio, Case report

## Abstract

Tuberculous (TB) disease remains an endemic pathology in Tunisia. the ileocecal region is the predominant site of involvement while gastroduodenal tuberculosis is very rare, this form is often presenting as one of the complications, mainly upper gastrointestinal stenosis or exceptionally as a perforation.

We describe a case of female patient aged 33 years-old presented with a 2-day history of acute abdominal pain, with a tenderness of the right hypochondrium and the epigastrium, ultrasound of the abdomen revealed gallbladder distension with a wall thickening.

The diagnosis of acute cholecystitis was suspected and the patient had an exploratory laparoscopy that revealed the presence of a perforated duodenal ulcer which was blocked by the gallbladder and several peri-duodenal lymph nodes. Cholecystectomy was performed and the edges of the ulcer were resected and the ulcer was sutured. Histological examination revealed duodenal tuberculosis and the patient was referred to the TB eradication program.

## Highlights

• The primary duodenal tuberculosis is very rare.
• The perforation is an exceptional complication its described for the second time in literature.
• The diagnosis is difficult with no clinical and endoscopic features.
• Surgical approach is recommended in complicated forms with antituberculos treatment.

## 1 Introduction and importance

Tuberculous (TB) disease remains an endemic pathology in Tunisia and according to the estimation published in the WHO Global Report, in 2017 the incidence rate was of 38/100,000 and the death rate of 0.04/100,000 [1].

For the gastrointestinal forms, the ileocecal region is the predominant site of involvement while gastroduodenal tuberculosis is very rare [2,3], this form is often presenting as one of the complications, mainly upper gastrointestinal stenosis or exceptionally as a perforation [4].

We report in this context a case of primary duodenal tuberculosis presenting as ulcer perforation with a review of the literature.

This case report has been reported in line with the SCARE 2020 Criteria [5].

## 2 Case presentation

A 33-year-old female patient with no significant past medical history, only a neglected epigastric pain under antacid medicine for 6 months with a slight undocumented weight loss, no history of TB, HIV infection, or diabetes, she was not a tobacco smoker and neither an alcoholic, the patient's family history was unremarkable.

She presented in the emergency room for acute abdominal pain, the physical examination found a patient in good general condition, feverish, with a tenderness of the right hypochondrium and the epigastrium. Laboratory analysis revealed a white blood cell count of 12,000/mm3 and microcytic hypochromic anemia. The chest x-ray was normal.

Ultrasound of the abdomen revealed gallbladder distension with a wall thickening and peri-cholecystic fluid.

**Fig. 1:**
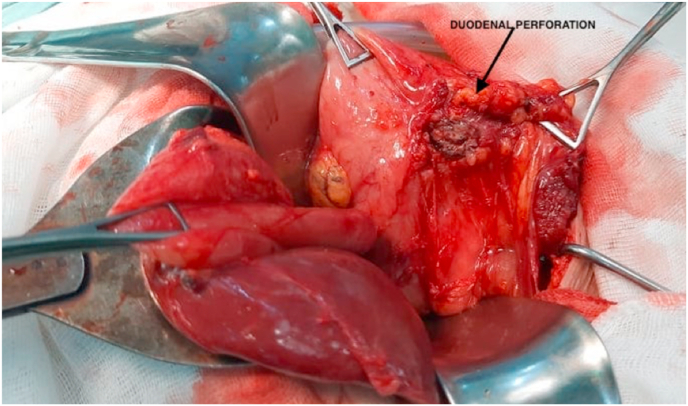
The duodenal perforation after the dissection of the gallbladder.

In view of these data the diagnosis of acute cholecystitis was suspected and the patient had an exploratory laparoscopy that revealed an attachment between the gallbladder and the duodenal bulb, several peri-duodenal lymph nodes, the largest of which was 2cm, Because of technical difficulties, a conversion to open surgery was decided. Then a careful dissection showed the presence of a perforated duodenal ulcer which was blocked by the gallbladder (Fig. 1). Cholecystectomy was performed and the edges of the ulcer were resected and the ulcer was sutured through separate stitches.

The postoperative follow-up was simple and the patient was discharged on day 5.

**Fig. 2:**
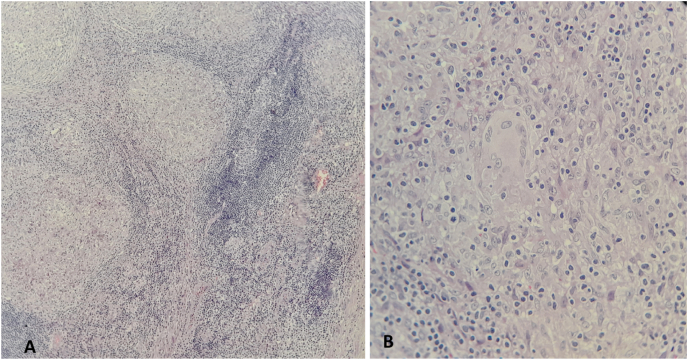
A-(HEx10): Lymphatic parenchyma altered by epithelioid granulomas B- (HEx40): Epithelioid granuloma with the presence of a giant cell Langhans type.

Histological examination of the ulcer showed that the duodenal mucosa heavily infiltrated by florid active inflammatory cells disrupting the glands, which consisted of neutrophils, lymphocytes, and plasma cells, no *Helicobacter pylori* or dysplasia. Examination of the peri-duodenal lymph node and the gallbladder lymph node showed caseating necrosis (Fig. 2), which confirmed the diagnosis of tuberculosis.

The patient was referred to the TB eradication program and quadritherapy (rifampin, isoniazid, ethambutol, pyrazinamide) was started and given subsequently for 6 months. The patient is doing well on follow-up with no recurrence.

## 3 Clinical discussion

Intestinal tuberculosis is the second most common form of gastrointestinal tuberculosis [6]. The site of predilection is the ileocecal region it concerns 42% of lesions of the digestive tract [7], explained by the existence of a physiological stasis at the level of the terminal ileum, the richness of this region in lymphoid elements, and the alkaline pH is favorable to the development of Koch's bacilli, yet duodenum is one of the rarest localization, even in the endemics areas [1]. The main reasons for gastroduodenal sparing are high acidity, a paucity of lymphoid tissue, and rapid transit of food in the stomach, albeit long-term therapy with H2 blockers can increase the incidence of gastroduodenal tuberculosis [4].

The diagnosis is often difficult due to the paucity of symptoms. Rao et al. in a review of 23 consecutive cases of gastroduodenal tuberculosis noted that vomiting (60.8%) and epigastric pain (56.5%) are the most common presenting symptoms [4].

With the absence of pathognomonic clinical or endoscopic features, complications are usually the most common cause of diagnosis, dominated by gastric outlet obstruction and gastrointestinal bleeding [8], while perforation is an exceptional complication. Although there were a few reported cases in the literature of gastric perforation [9], to our knowledge there was to date data only one reported observation of duodenal perforation secondary to duodenal tuberculosis [10].

Our case report is the second one to document this type of complication due to the rarity of the form of duodenal tuberculosis and the difficulty of diagnosis.

In the early stages, there are no pathognomic features, endoscopy may reveal duodenal bulb deformity and biopsy has a poor yield even in ulcerated lesions, it rarely reveals granulomas and often shows non-specific inflammatory lesion, Guilinsky et al. in a review of 27 patient who underwent endoscopic biopsies of duodenal tuberculosis, 20 had nonspecific lesions and granulomas were found in only 7 patients [11]. Koch bacilli are rarely recovered from the biopsy material it's mainly due to the failure of routine endoscopic biopsies to include the submucosa which most predominant location of the lesions [12,13].

A chest x-ray may show evidence of pulmonary TB in up to 20% of cases, in our patient the x-ray was normal.

The CT-scan may show a thickening of the gastric or duodenal wall, associated with enlarged local lymph nodes, it may be the only clue to diagnosis [4].

The predominant complication is obstruction and perforation is rare because of the surrounding inflammatory fibrosis induced by the ulcer.

This difficulty of diagnosis and the absence of specificity of endoscopic and radiological features may explain why most patients must undergo surgery before confirming the disease. However, surgery is not indicated as a first-choice procedure in chronic, uncomplicated cases, because intestinal tuberculous lesions often regress or disappear under appropriate antituberculous medication.

In the case of our patient, the duodenal perforation was blocked by the gallbladder that explains the absence of peritonitis like in the case reported by Berney et al. [10], but the surgical indication was maintained due to suspicion of acute cholecystitis. Furthermore, the histological examination of the peri-duodenal lymph node confirmed the diagnosis by showing caseating necrosis, like often reported in the literature [12], which allowed the start of proper antituberculous therapy.

## 4 Conclusion

Although it is uncommon, duodenal tuberculosis should be considered in patients with gastric symptoms or lesions of unknown etiology, and it should be kept in mind even in the case of negative biopsies. Patients presenting a diagnostic dilemma or with complications require surgery.

## Sources of funding

No sources of funding.

## Ethical approval

There is no ethical committee in our country (Not applicable for this Manuscript).

## The consent statement for publication

Written informed consent was obtained from the patient for publication of this case report and accompanying images. A copy of the written consent is available for review by the Editor-in-Chief of this journal on request.

## Author statement

All authors contributed to the study design.

All authors read and approved the final manuscript.

## Registration of research studies

This is not a research it's a cases report.

## Guarantor

Atri Souhaib.

## Provenance and peer review

Not commissioned, externally peer reviewed.

## Declaration of competing interest

All authors declare that they have no any conflicts of interest.
